# Invasive pulmonary aspergillosis in three cases of influenza

**DOI:** 10.1002/hsr2.578

**Published:** 2022-04-01

**Authors:** Glória Selegatto, Anna C. Turdo, Izabel Marcilio, Yeh‐Li Ho

**Affiliations:** ^1^ Department of Infectious and Parasitic Diseases Hospital das Clinicas da Faculdade de Medicina da Universidade de Sao Paulo Sao Paulo Brazil; ^2^ Epidemiological Surveillance Department Hospital das Clinicas da Faculdade de Medicina da Universidade de Sao Paulo Sao Paulo Brazil

**Keywords:** influenza, invasive pulmonary aspergillosis

## 1 BACKGROUND

Influenza infection with or without bacterial coinfection agents are well described, contributing to higher severity and mortality.^1^ Classically, invasive pulmonary aspergillosis (IPA) is described in immunocompromised patients, such as neutropenic or organ transplant recipients. However, recent reports on influenza‐associated IPA in patients without classical risk factors have been published. Thus, the presence of *Aspergillus* spp. in cultures could be interpreted as colonizers, resulting in a challenging diagnosis.^2^ An European cohort described 19% influenza patients with IPA, but there is no information about that condition in Latin America.^3^

In this study, we describe three cases of IPA in severely ill influenza‐infected patients in a Tertiary University Hospital in São Paulo, Brazil (Table 1).

**Table 1:** Clinical and laboratory findings

Case	Baseline conditions	Symptoms until hospitalization	Viral agents	Other agents	Previous corticoid therapy	Radiologic exam	C‐reactive protein (mg/L)	Leukocytes (cells/mm³)	Introduction/Duration of antifungal therapy	Hospitalization time/Outcome
1	HIV Asthma	7 days	Influenza A H1N1	None	Yes	X‐ray Bilateral Interstitial infiltrate	124	28,710	D2/5 days	7 days/Death
2	Pulmonary Tuberculosis Malnutrition	10 days	Influenza A H1N1	*Mycobacterium tuberculosis*	Yes	CT Cavitation Micronodules Consolidation	262	9040	D3/7 days	30 days/Death
3	Former cocaine user Congestive heart failure	20 days	Influenza A H1N1 Influenza A Non H1N1	*Klebsiella pneumoniae*	No	CT Interstitial infiltrate “Ground glass” Consolidation foci	264	5730	D16/18 days	38 days/Death

*Note*: Reference values: Leukocytes/mm³ ‐ 4000–11000; C‐reactive protein (CPR) mg/L ‐ <5 mg/L.

## 2 CASE REPORT

### 2.1 Case 1

Female, 47 years old, asthma, HIV since 2010 with undetectable HIV‐viral load and 195 CD4+. She presented with fever, cough, and dyspnea for 4 days being prescribed levofloxacin and prednisone in another hospital due to suspected bacterial pneumonia. We admitted her 3 days later with acute respiratory failure with a positive Influenza test. Oseltamivir, ceftriaxone, and cotrimoxazole for *Pneumocystis pneumonia* were started. Mechanical ventilation (MV) and intensive care unit (ICU) were required since admission. Bronchoalveolar lavage (BAL) was performed and resulted positive for both Influenza A H1N1 and *Aspergillus* sp. culture. Amphotericin lipid complex was started in D2 of hospitalization. She evolved with renal impairment, dialysis, need for vasoactive drugs, and neuromuscular block due to bronchospasm. She died at D7.

### 2.2 Case 2

**Figure 1:**
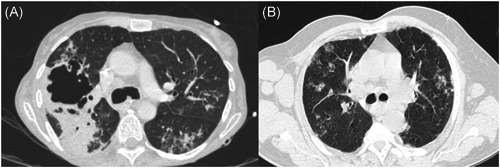
Radiologic images from four patients with influenza and aspergillosis. (A) Case 2; (B) Case 3.

Female, 53 years old, no previous condition, with 2‐month duration loss of weight, dyspnea, and cough presented in outpatient medical care. She performed a CT‐scan and returned for appointment after 10 days with acute respiratory failure. She was immediately hospitalized and tested positive for influenza. MV support and vasoactive drug were required (Figure 1A). A bronchoscopy was performed and BAL resulted positive for *Mycobacterium tuberculosis*, Influenza A H1N1 virus, and *Aspergillus* sp. Amphotericin lipid complex and treatment tuberculosis regime (TB) were initiated. After 10 days, she presented with a megacolon due to a TB infection. She continued with positive bacilloscopic until D22 and died on D30.

### 2.3 Case 3

Male, 33 years old, previous no‐treatment cardiac congestive failure, former cocaine user. He presented with 20 days of cough, hemoptysis, night sweating, and dyspnea. The first hypothesis was bacterial pneumonia and he received oral antibiotics with no improvement after therapy. The patient evolved with respiratory failure and was admitted to the emergency room with suspected influenza (Figure 1B). He needed MV and a bronchoscopy was performed after 1 day. His BAL resulted positive for Influenza A H1N1 and oseltamivir was associated. Due to his poor evolution, a new BAL was performed after 14 days and became positive to Influenza A Non H1N1, and the culture identified *Aspergillus* sp. and *Klebsiella pneumoniae* resistant to carbapenems. Meropenem, amikacin, and amphotericin lipid complex were started. After 34 days, he presented with massive hemoptysis and died. A necropsy was performed and invasive aspergillosis was diagnosed in a histopathologic study.

## 3 DISCUSSION

In our Hospital, from January to October‐2018, 280 cases of severe respiratory syndrome were notified, 136 cases in adult inpatients, and 85 cases of viral respiratory infection were admitted to the hospital. Influenza A virus was detected in 76 patients, while other respiratory viruses were detected in nine patients (Adenovirus, Parainfluenza 3, and Rhinovirus).

*Aspergillus* spp. comprehend a genus of ubiquitous saprophyticus mold disperse in the environment and can colonize the respiratory epithelium of human beings. Considering its capability to cause invasive disease in critically ill patients, mainly immunocompromised hosts, the identification in respiratory specimens represents a challenge in the clinical management of these patients.^4^

The EORTC/MSG states the diagnosis criteria to IPA and evaluates three variables: host factors, radiologic findings, and microbiologic findings. Host factors encompass only immunocompromised patients due to: (1) 3 or more weeks on corticosteroid therapy, (2) hematological malignancies or prolonged neutropenia, and (3) immunobiological therapy.^5^, ^6^

*Aspergillus*‐influenza coinfection has been described since 1952, with few reports until 2009.^7^, ^8^ However, after Influenza A H1N1 pandemic, several cases were observed especially in critically ill patients. Despite not being included as a classical immunosuppressive condition, the influenza virus infection may cause damage and impaired immune response to the mucosal respiratory epithelium assisting the invasive disease by previously colonizing *Aspergillus* spp., prompting new criteria including the influenza infection as a host factor, not limited to just immunocompromised patients.^3^

Based on these findings, a retrospective cohort during the 2009–2016 period conducted in Belgium and The Netherlands concluded that Influenza A and E infections were an independent variable associated with pulmonary aspergillosis and a risk factor for mortality in that population.^2^

In our review, most cases have been reported by North America and Europe, with no reports from Latin America. A reason to explain this issue is the poor access to diagnostic methods in most countries and locations from Latin America.

Another reason is that, although our hospital is a reference to severe influenza infection cases since 2009, there is no early routine algorithm to suspect IPA in nontransplanted patients or patients without hematological malignancies.

In our case reports, the initial diagnosis was performed by LBA culture in the first two cases and a proven diagnosis was performed by necropsy in the last case. Despite the efforts made, all cases resulted in death. Some hypotheses to explain these outcomes are possible.

All patients had previous corticoid use. This is a known risk factor in patients with influenza and other infections to evolve to severe coinfections and aspergillosis.^2^, ^3^ Additionally, all patients used lipid complex amphotericin while *Aspergillus* treatment guidelines recommend voriconazole or liposomal amphotericin (Lamb) as first‐choice therapy for IPA.^9^, ^10^ Due to lack of these drugs in our hospital, lipid complex amphotericin was prescribed in these cases possibly contributing to the negative outcomes shown by our case reports.

## 4 CONCLUSIONS

These were the first cases of influenza‐associated aspergillosis described in Brazil and Latin America.

This case report is important to show that this illness exists in Brazil and should be explored in severe influenza and other respiratory virus infection, in patients with no classic baseline condition, due to its severity and high risk of mortality.

## AUTHOR CONTRIBUITIONS

*Conceptualization*: Glória Selegattto, Anna Cláudia Turdo, and Yeh‐Li Ho. *Investigation*: Glória Selegatto and Izabel Marcilio*. Supervision*: Izabel Marcilio and Yeh‐Li Ho. *Writing—review, and editing*: Gloria Selegatto and Yeh‐Li Ho. *Writing*—*original draft*: Gloria Selegatto. All authors have read and approved the final version of the manuscript. The corresponding author, Gloria Selegatto, had full access to all of the data in this study and takes complete responsibility for the integrity of the data and the accuracy of the data analysis.

## CONFLICTS OF INTEREST

The authors declare no conflicts of interest.

## Data Availability

The data that support the findings of this study are available on request from the corresponding author. The data are not publicly available due to privacy or ethical restrictions (medical records data).

## References

[1] Martin‐Loeches I , Schultz MJ , Vincent J‐L , et al. Increased incidence of co‐infection in critically ill patients with influenza. Intensive Care Med. 2017;43:48‐58. 10.1007/s00134-016-4578-y 27709265

[2] Huang L , Zhang N , Huang X , et al. Invasive pulmonary aspergillosis in patients with influenza infection: a retrospective study and review of the literature. Clin Respir J. 2019;13(4):202‐211. 10.1111/crj.12995 30661296

[3] Schauwvlieghe AFAD , Rijnders BJA , Philips N , et al. Invasive aspergillosis in patients admitted to the intensive care unit with severe influenza: a retrospective cohort study. Lancet Respir Med. 2018;6(10):782‐792. 10.1016/s2213-2600(18)30274-1 30076119

[4] Thompson GR , Young J‐AH . Aspergillus infections. N Engl J Med, 385:1496‐1509. https://doiorg/101056/NEJMra2027424 10.1056/NEJMra2027424 34644473

[5] Blot SI , Taccone FS , Van den Abeele AM , et al. A clinical algorithm to diagnose invasive pulmonary aspergillosis in critically ill patients. Am J Respir Crit Care Med. 2012;186(1):56‐64. 10.1164/rccm.201111-1978OC 22517788

[6] Donnelly JP , Chen SC , Kauffman CA , et al. Revision and update of the consensus definitions of invasive fungal disease from the European Organization for Research and Treatment of Cancer and the Mycoses Study Group Education and Research Consortium. Clin Infect Dis. 2019;71:1367‐1376. 10.1093/cid/ciz1008 PMC7486838 31802125

[7] Abbott JD , Fernando HV , Gurling K , Meade BW . Pulmonary aspergillosis following post‐influenzal bronchopneumonia treated with antibiotics. Br Med J. 1952;1(4757):523‐525. 10.1136/bmj.1.4757.523 14904967 PMC2022929

[8] Shah MM , Hsiao EI , Kirsch CM , Gohil A , Narasimhan S , Stevens DA . Invasive pulmonary aspergillosis and Influenza co‐infection in immunocompetent hosts: case reports and review of the literature. Diagn Microbiol Infect Dis. 2018;91(2):147‐152. 10.1016/j.diagmicrobio.2018.01.014 29454654 PMC5970059

[9] Patterson TF , Thompson GR , Denning DW , et al. Practice guidelines for the Diagnosis and Management of Aspergillosis: 2016 Update by the Infectious Diseases Society of America. Clin Infect Dis. 2016;63(4):e1‐e60. 10.1093/cid/ciw326 27365388 PMC4967602

[10] Ullmann AJ , Aguado JM , Arikan‐Akdagli S , et al. Diagnosis and management of Aspergillus diseases: executive summary of the 2017 ESCMID‐ECMM‐ERS guideline. Clin Microbiol Infect. 2018;24(Suppl 1):e1‐e38. 10.1016/j.cmi.2018.01.002 29544767

